# Vitamin A Status Is More Commonly Associated With Symptoms and Neurodevelopment in Boys With Autism Spectrum Disorders—A Multicenter Study in China

**DOI:** 10.3389/fnut.2022.851980

**Published:** 2022-04-05

**Authors:** Ting Yang, Li Chen, Ying Dai, Feiyong Jia, Yan Hao, Ling Li, Jie Zhang, Lijie Wu, Xiaoyan Ke, Mingji Yi, Qi Hong, Jinjin Chen, Shuanfeng Fang, Yichao Wang, Qi Wang, Chunhua Jin, Jie Chen, Tingyu Li

**Affiliations:** ^1^Chongqing Key Laboratory of Childhood Nutrition and Health, Children's Hospital of Chongqing Medical University, Ministry of Education Key Laboratory of Child Development and Disorders, National Clinical Research Center for Child Health and Disorders, Chongqing, China; ^2^Department of Developmental and Behavioral Pediatrics, The First Hospital of Jilin University, Changchun, China; ^3^Department of Pediatrics, Tongji Hospital, Tongji Medical College, Huazhong University of Science and Technology, Wuhan, China; ^4^Department of Children Rehabilitation, Hainan Women and Children's Medical Center, Haikou, China; ^5^Children Health Care Center, Xi'an Children's Hospital, Xi'an, China; ^6^Department of Children's and Adolescent Health, Public Health College of Harbin Medical University, Harbin, China; ^7^Child Mental Health Research Center of Nanjing Brain Hospital, Nanjing, China; ^8^Department of Child Health Care, The Affiliated Hospital of Qingdao University, Qingdao, China; ^9^Maternal and Child Health Hospital of Baoan, Shenzhen, China; ^10^Department of Child Healthcare, Shanghai Children's Hospital, Shanghai Jiao Tong University, Shanghai, China; ^11^Children's Hospital Affiliated of Zhengzhou University, Zhengzhou, China; ^12^NHC Key Laboratory of Birth Defect for Research and Prevention, Hunan Provincial Maternal and Child Health Care Hospital, Changsha, China; ^13^Deyang Maternity and Child Healthcare Hospital, Deyang, Sichuan, China; ^14^Department of Children Health Care, Capital Institute of Pediatrics, Beijing, China

**Keywords:** autism spectrum disorder, vitamin A, neurodevelopment, clinical symptoms, sex differences

## Abstract

**Background:**

Autism spectrum disorder (ASD) is a neurodevelopmental disorder, and show a striking male bias in prevalence. Vitamin A (VA) is essential for brain development, and abnormalities in its metabolite retinoic acid are associated with the pathophysiology of ASD. This national multicenter study was conducted to investigate the relationship between serum VA level and core symptoms in ASD children and whether there are still sex differences.

**Method:**

A total of 1,300 children with ASD and 1,252 typically-developing (TD) controls aged 2–7 years old from 13 cities in China were enrolled in this study. The symptoms of children with ASD were evaluated by the Autism Behavior Checklist (ABC), Social Responsiveness Scale (SRS), and Childhood autism rating scale (CARS). The neurodevelopmental level of the children was evaluated with the revised Children Neuropsychological and Behavior Scale (CNBS-R2016). The serum level of VA was measured by high-performance liquid chromatography (HPLC).

**Results:**

The serum VA level in children with ASD was significantly lower than that in TD children, especially in boys with ASD. Furthermore, VA levels in male children with ASD were lower than those in female children with ASD. In addition, we found that serum VA level was negatively correlated the SRS, CARS and communication warming behavior of CBNS-R2016 scores in boys with ASD. In terms of developmental quotients, serum VA level was positively associated with the general quotient, language quotient, gross motor quotient and personal-social quotient of boys with ASD, but no difference was found in girls with ASD.

**Conclusions:**

ASD children, especially boys, have lower serum VA levels than TD children. Moreover, serum VA status is more commonly associated with clinical symptoms and neurodevelopment in boys with ASD.

## Introduction

Autism spectrum disorder (ASD) is a developmental disability characterized by persistent impairments in social interaction and the presence of restricted, repetitive patterns of behaviors, interests, or activities (1). The overall prevalence of ASD in 8-year-old children is 23.0/1,000 (one in 44) in the United States (2), and in China it has been estimated to be 7.0/1,000 among 6–12-year-old children (3), with an increasing trend over time. As ASD is a lifelong disability, such increasing trend has brought an enormous burden on family and society and increased health care costs. There is a sex-bias in ASD children, ASD diagnosis is approximately 4.2 times more common among boys than girls (2). Accumulating evidence suggests that the clinical core symptoms of ASD children are inconsistent between different sexes. Boys with ASD were found to exhibit higher levels of repetitive and stereotyped behaviors than girls (4–7). More recently, studies have paid attention to the differences in blood biomarkers and metabolites between ASD children of different sexes (8, 9).

Vitamin A (VA) is essential for brain development and is transported in the blood as retinol and functions in various processes as its metabolite retinoic acid (RA) in tissues (10). RA plays prominent roles in mediating neuron differentiation, synaptic plasticity and tissue formation by activating gene transcription as a ligand for the transcription factor RA receptors (RARs) (11). Genetic deletions in mice revealed that RA signaling *via* the retinoic acid receptors RXRG and RARB and CYP26B1-dependent catabolism is associated with appropriate molecular patterns in the prefrontal and motor areas, and the development of the prefrontal cortex-medial dorsal thalamus connection, which are thought to be altered in ASD (12). Moreover, other studies suggest that VA can increase oxytocin (OXT) levels in ASD patients through RA-RAR signaling and the CD38-OXT signaling pathway, and brain activity and social skills in autistic patients may be significantly increased through OXT (13–15). Our previous clinical study showed that VA supplementation for 6 months effectively increased serum VA concentration and the expression levels of proteins involved in the RARβ-CD38-OXT axis, and reducing social dysfunction in children with ASD (16, 17).

Vitamin A deficiency (VAD) is still considered public health problem, particularly in some developing countries, with an estimated 19 million pregnant women and 140 million children suffering from VAD worldwide (18). VA is essential for brain and neural system developments begin at the embryonic stage. Our previous study found that the serum VA levels in ASD children were significantly lower than that in control children. Interestingly, we further observed a positive association between maternal micronutrients supplementation and serum VA levels in ASD children, low serum VA levels in ASD children were associated with significantly lower rates of multivitamin supplementation during pregnancy (19). We hypothesize that VAD in ASD children may begin at the embryonic stage. Accumulating evidence, including the sex-specific effect of biochemical parameters, indicates that the ASD-specific behavioral alterations are differentially regulated in boys and girls. However, no studies have been conducted to date to investigate the differences in serum VA levels between children of different sexes and their association with the core symptoms of ASD. Accordingly, the present study used a nationwide multicenter cross-sectional survey to determine the differences in serum VA levels between ASD children of different sexes, as well as the association between the VA status and core symptoms of ASD in children.

## Methods

### Study Design and Ethical Aspects

This is a national cross-sectional clinical study of 2–7-year-old children in China conducted from May 2018 to December 2019. The study was prospectively sponsored by the Subspecialty Group of Developmental and Behavioral Pediatrics, the Society of Pediatrics, and the Chinese Medical Association, with the participation of 13 members of the China Autism Clinical Research Alliance. The study was approved by the ethics committee of the Children's Hospital of Chongqing Medical University, Approval Number: (2018) IRB (STUDY) NO.121, and registered in the Chinese Clinical Trial Registry (Registration number: ChiCTR2000031194). Participation in this research was voluntary, and parents provided informed consent forms for all participants.

### Study Participants

**Figure 1:**
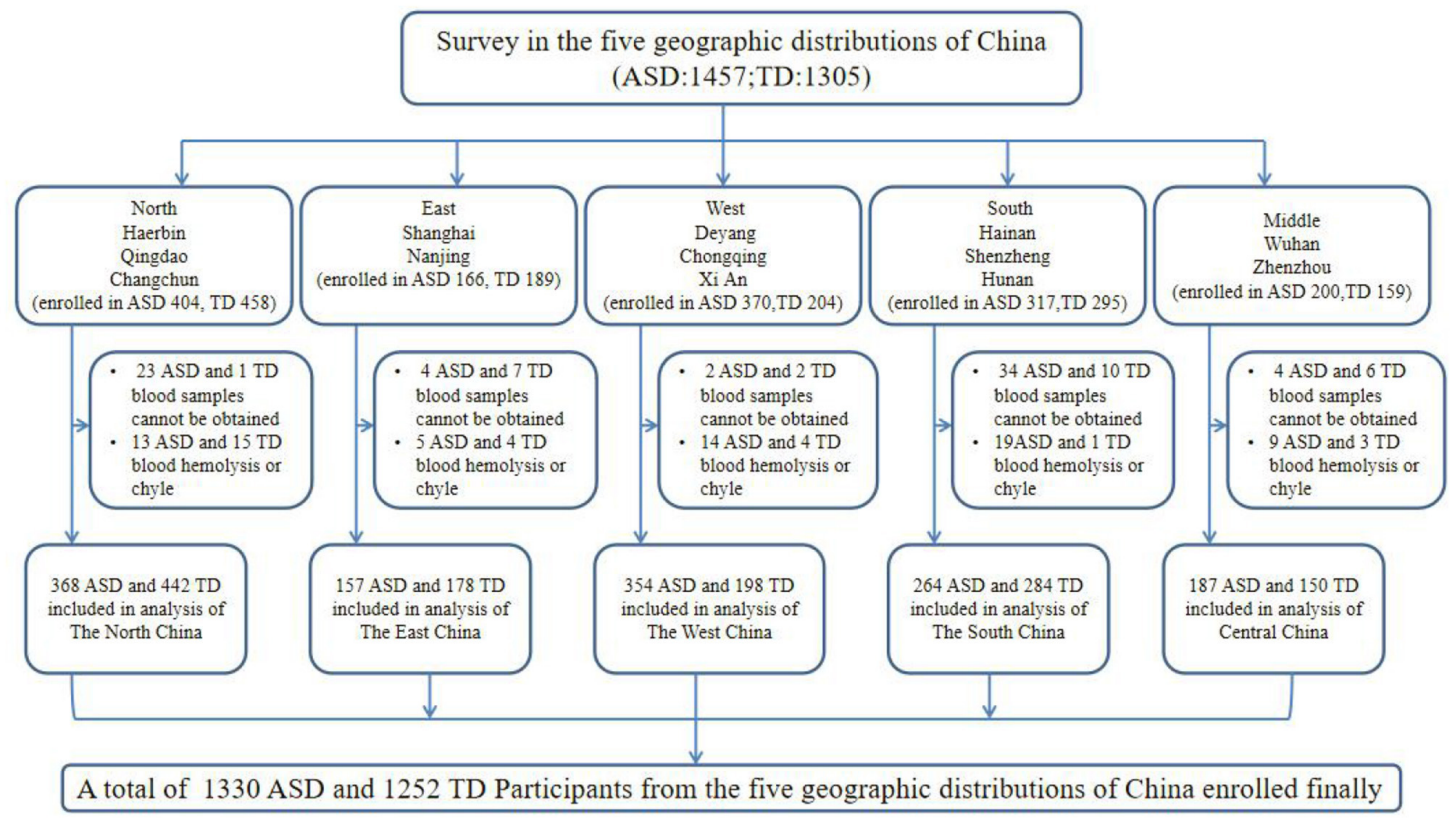
Flow diagram illustrating the survey participants: a multi-center study in China. ASD, autism spectrum disorder; TD, typically developing.

The study population was recruited from 13 locations in five geographical regions of China, namely the North (Harbin, Qingdao, and Changchun), East (Shanghai and Nanjing), West (Chongqing, Deyang, and Xi'an), South (Shenzhen, Haikou, and Changsha), and Middle (Wuhan and Zhengzhou). The study profile is illustrated in Figure 1. A total of 2,552 participants (1,300 2~7-year-old children with ASD and 1,252 typically-developing (TD) control children) were enrolled in this study. ASD children from hospital rehabilitation department or developmental behavioral pediatrics and rehabilitation training institutions were included in this study. The inclusion criteria for participants in the ASD group were the following: 1) 2–7 years old at enrollment and 2) have a diagnosis of ASD documented in their medical history [The diagnoses were conducted by an experienced psychologist or an experienced developmental pediatrician at the Children's Hospital, based on the criteria for autism defined in the Diagnostic and Statistical Manual of Mental Disorders, Fifth Edition (DSM-5)]. We also recruited participants for the TD group from online volunteers and local preschools who met the following inclusion criteria: (1) absence of ASD diagnosis and (2) no family history of ASD in first- and second-degree relatives. The exclusion criteria for participants in the ASD group were the following: (1) presence of a genetic or neurological disorder of known etiology (*e.g*., Fragile X, Rett's syndrome), (2) significant sensory or motor impairment, (3) major physical/medical problems, (4) seizures, (5) history of a serious head injury, and (6) an acute or chronic infection in the previous 3 months. The exclusion criteria for participants in the TD group were the following: (1) significant medical or neurological conditions affecting growth, development or cognition (*e.g*., CNS infection, seizure disorder, diabetes, tuberous sclerosis) or sensory impairments, such as significant vision or hearing loss and (2) had a history of language impairments, or social developmental disorders.

### Clinical Measures of ASD Children

Information on demographics (*e.g*., name, age, and sex), parent education level and family income were collected through face-to-face interviews with parents of the ASD and control children conducted by investigators who had received comprehensive professional training. The symptoms of the ASD children were assessed with two parent-report instruments, namely the Aberrant Behavior Checklist (ABC; in which the score of TD children should be <53) (20), the Social Responsiveness Scale (SRS; in which the score of TD children should be <65) (21), and one developmental pediatrician observation instruments, namely the Childhood Autism Rating Scale (CARS; in which the score of TD children should be <30) (22). The neurodevelopmental level of the children was evaluated with the revised Children Neuropsychological and Behavior Scale (CNBS-R2016), which has been revised and widely used in China. CNBS-R2016 includes five separate sub-scales, namely Gross Motor, Personal-Social, Language, Fine Motor, Adaptive Behavior, and an independent sub-scale (Communication Warning Behavior) was added to assess autism symptoms. A general quotient or subscale developmental quotient <70 points [ <2 standard deviations (SDs)] indicates developmental delay (DD). For the sub scale of communication warning behavior, a score of <7 points indicates typical development. Follow up is required between 7 and 12 points; scores from 12 to 30 points indicate the risk of communication and interaction barriers; a score >30 indicates high suspicion of ASD. As Li et al. reported, the effectiveness of developmental assessment in children with ASD by the CNBS-R2016 was consistent with that of the Griffiths Mental Development Scales for China (GDS-C) (23). In this study, the valid evaluation sample sizes of ABC, SRS and CARS were 1,100, 1,110, and 1,119, respectively. The valid evaluation sample size of CNBS-R2016 was 957.

### Laboratory Measurements

A 5 ml aliquot of venous blood was collected from the participants by venipuncture, which was immediately centrifuged at 3,000 rpm at room temperature for 10 min to obtain the serum. The serum was then used for detecting the level of VA (retinol) by high performance liquid chromatography (HPLC) according to previously described methods (24). Briefly, serum (200 ul) was deproteinated with 200 ul dehydrated alcohol. The VA was extracted from the serum by hexane (1,000 ul). Then, 500 ul of supernatant was extracted and the hexane in it was evaporated with nitrogen gas. A mobile phase mixture (methanol: water = 97:3) was used to dissolve the VA residue. Then, the prepared sample was detected by HPLC on a Shimadzu DGU-20 As Prominence HPLC System (Shimadzu Corporation, Kyoto, Japan) at 315 nm, using a C18 column. The entire procedure was performed by the same operator in a dark room to protect the serum from light. Serum VA concentrations of >0.3, 0.2–0.3, and <0.2 mg/L were defined as VA normal (VAN), marginal VAD (MVAD), and VAD, respectively (25).

### Statistical Analyses

The Kolmogorov-Smirnov goodness-of-fit test was used to test the distribution of each data set for normality before analysis. Data were analyzed using the SPSS software version 22.0 (IBM Corporation, Armonk, NY, USA). The mean and standard deviation (SD) for normal continuous variables and the frequency and percentage per category for categorical variables were used to analyze the demographic characteristics of children in each group (the TD group *vs*. ASD group). Group differences were compared by independent *t*-test of continuous variables and chi square test or Fisher's exact test of categorical variables. Univariate and multivariate (adjusted for age) logistic regression models were used to compare VA levels between the TD children and the ASD children, as well as the VA levels of TD children and ASD children of different sexes and the VA levels of ASD children of different sexes. We further compared the effect of VA on the total and subscale scores in ASD groups using the Multivariate Linear Regression Models for boys and girls separately, with age as a covariate. Odds ratio (OR) and 95% confidence interval (CI) were evaluated to determine associations. A two-sided *p*-value < 0.05 is regarded as statistically significant.

## Results

### Comparison of Demographic Characteristics Between TD and ASD Groups

As shown in Table 1, a total of 1,252 TD children and 1,300 children with ASD were enrolled in this study. The median interquartile range (IQR) age of the TD group was 4.39 (3.36–5.34) years, including 822 boys and 430 girls. The ASD children median IQR age was 3.97 (3.15–4.90) years, with 1,088 boys and 242 girls. There were statistically significant differences in age, sex and residence between the two groups (*P* < 0.001). The parents' educational levels in the TD group were higher than that in the ASD group (*P* < 0.001). All the Z-scores (Z_HA_, Z_WA_, Z_BMIA_) showed no significant differences between TD children and ASD children.

**Table 1:** Demographic characteristics of the participants in TD and ASD groups.

**Variable**	**TD (*N* = 1,252)**	**ASD (*N* = 1,330)**	**Z/T/chi-square**	***P***
**Gender**, ***n*** **(%)**				
Boys	822 (65.655)	1,088 (81.805)	χ^2^ = 87.364	<0.001
Girls	430 (34.345)	242 (18.195)		
Age (years), Median (IQR)	4.390 (3.360–5.340)	3.970 (3.145–4.900)	Z = −5.789	<0.001
Father's educational levels, *n* (%)				
Middle school or below	143 (12.181)	259 (20.394)	χ^2^ = 45.991	<0.001
High school	204 (17.376)	276 (21.732)		
College or above	827 (70.443)	735 (57.874)		
Mother's educational levels, *n* (%)				
Middle school or below	142 (12.014)	301 (23.516)	χ^2^ = 106.904	<0.001
High school	185 (15.651)	309 (24.141)		
College or above	855 (72.335)	670 (52.344)		
Residential place, *n* (%)				
Urban	1,043 (89.145)	964 (75.549)	χ^2^ = 76.621	<0.001
Rural	127 (10.855)	312 (24.451)		
Boys	*N* = 797	*N* = 904		
Z_HA_, mean ± SD	0.120 ± 1.130	0.223 ± 1.167	T = −1.844	0.065
Z_WA_, mean ± SD	0.322 ± 1.121	0.393 ± 1.156	T = −1.287	0.198
Z_BMIA_, mean ± SD	0.365 ± 1.173	0.370 ± 1.374	T = −0.083	0.934
Girls	*N* = 413	*N* = 198		
Z_HA_, mean ± SD	0.051 ± 1.148	0.228 ± 1.104	T = −1.802	0.072
Z_WA_, mean ± SD	0.199 ± 1.038	0.249 ± 1.151	T = −0.536	0.592
Z_BMIA_, mean ± SD	0.243 ± 1.128	0.141 ± 1.323	T = 0.987	0.324

*ASD, autism spectrum disorder; TD, typically developing; IQR, inter-quartile range.*

*Z_HA_, Z-scores for height; Z_WA_, Z-scores for weight; Z_BMIA_, Z-scores for BMI.*

*Data was shown as Median (IQR) or number (percentage). Chi-square test and Mann-Whitney U test were used in the analysis*.

### Serum VA Levels in TD and ASD Group

To determine whether there are differences in serum VA levels in children with ASD and TD, we measured the levels of serum VA in the two groups. The results of the univariate and multivariate binary logistic regression (adjusting for age) analysis are shown in Table 2. The univariate binary logistic regression analysis showed that the serum VA levels in ASD children were significantly lower than that in TD children (*OR* = 0.315, 95% CI: 0.111 to 0.897, *P* < 0.05). Taking age as a covariate, the multivariate binary logistic regression analysis showed that there was still significant difference in VA level between the two groups (*OR* = 0.275, 95% CI: 0.096 to 0.789, *P* < 0.05).

**Table 2:** Comparison of the levels of serum VA between children with ASD and TD.

**Group**	**VA Mean (SD)**	**Unadjusted**	***P***	**Adjusted**	***P***
		**OR (95% CI)**		**OR (95% CI)**	
TD (*N* = 1,252)	0.353 (0.074)	reference	0.03	reference	0.016
ASD (*N* = 1,330)	0.347 (0.073)	0.315 (0.111 to 0.897)		0.275 (0.096 to 0.789)	

*TD, typically developing; ASD, autism spectrum disorder; OR, odds ratio; CI, confidence interval.*

*Uni-variate Logistics Regression was used for unadjusted model and Binary Logistics Regression was used for adjusted model (adjusted for age)*.

### Comparison of VA Levels by Group for Children of Different Sexes

We further analyzed differences in serum VA levels between the TD and ASD children in boys and girls groups using the binary logistics model. The univariate and multivariate (adjusting for age) binary logistic regression analysis showed that the VA levels in ASD children were significantly lower than that in TD children in boys (OR = 0.207, 95% CI: 0.062 to 0.693, *P* < 0.05; OR = 0.190, 95% CI:0.056 to 0.639, *P* < 0.05, respectively), but there was no difference between the two groups in girls. Additionally, VA levels of ASD children in boys were lower than those in girls (OR = 17.214, 95% CI: 2.591 to 114.375, *P* < 0.05; OR = 16.559, 95% CI: 2.471 to 110.959, *P* < 0.05, respectively) (Table 3).

**Table 3:** Comparison of the levels of serum VA between children with ASD and TD in different gender.

**Group**	**VA mean(SD)**	**Unadjusted**	***P***	**Adjusted**	***P***
		***OR* (95% CI)**		***OR* (95% CI)**	
**Boys**					
TD (*N* = 822)	0.353 (0.076)	reference	0.011	reference	0.007
ASD (*N* = 1,087)	0.344 (0.075)	0.207 (0.062 to 0.693)		0.190 (0.056 to 0.639)	
**Girls**					
TD (*N* = 430)	0.354 (0.071)	reference	0.294	reference	0.441
ASD (*N* = 244)	0.360 (0.069)	3.343 (0.351 to 31.794)		2.443 (0.252 to 23.665)	
**Gender**					
Boys with ASD (*N* = 1,087)	0.344 (0.075)	reference	0.003	reference	0.004
Girls with ASD (*N* = 244)	0.360 (0.069)	17.214 (2.591 to 114.375)		16.559 (2.471 to 110.959)	

*TD, typically developing; ASD, autism spectrum disorder. OR, odds ratio; CI, confidence interval.*

*Uni-variate Logistics Regression was used for unadjusted model and Binary Logistics Regression was used for adjusted model (adjusted for age)*.

### Effect of the Serum VA Level on the Scores of ABC, SRS, CARS and Communication Warning Behavior of CBNS-R2016 in the ASD Children in Different Sex Groups

We used a linear regression model to analyze the effect of the serum VA level on the scores of ABC, SRS, CARS, and communication warning behavior of CBNS-R2016 scales, with age as a covariant. The results revealed that VA level was negatively associated with the social awareness subscale scores of SRS (β = −0.065, 95% CI: −5.478 to −0.243, *P* < 0.05), the social cognition subscale scores of SRS (β = −0.066, 95% CI: −7.356 to −0.411, *P* < 0.05), the social communication subscale scores of SRS (β = −0.107, 95% CI: −19.274 to −5.611, *P* < 0.05), the SRS total score (β = −0.090, 95% CI: −43.795 to −9.113, *P* < 0.05), the CARS total scores (β = −0.126, 95% CI: −17.000 to −6.182, *P* < 0.05), and the scores of communication warming behavior of CBNS-R2016 (β = −0.080, 95% CI: −41.721 to −5.012, *P* < 0.05). We further performed stratified analysis by sex and found that the serum VA level was negatively correlated with the SRS scores, the CARS scores and the communication warming behavior of CBNS-R2016 scores in boys, while the serum VA level was only negatively associated with CARS score in girls. The differences in each subscale for both sex groups are detailed in Table 4.

**Table 4:** Effect of serum VA level on the scores of ABC, SRS, CARS and communication warming behavior of CBNS-R2016 in the ASD children at different gender groups.

**Scale scores**	**Boys**	***P***	**Girls**	***P***	**Total**	***P***
	**β (95% CI)**		**β (95% CI)**		**β (95% CI)**	
ABC scale scores	*n* = 895		*n* = 205		*n* = 1,100	
Sensory	0.040 (−1.865 to 7.589)	0.235	−0.102 (−18.562 to 3.068)	0.159	0.017 (−3.115 to 5.512)	0.586
Relating	−0.023 (−8.864 to 4.336)	0.501	−0.044 (−20.943 to 10.985)	0.539	−0.026 (−8.681 to 3.466)	0.400
Body and object use	0.048 (−1.767 to 10.949)	0.157	−0.108 (−28.857 to 3.785)	0.131	0.018 (−4.161 to 7.715)	0.557
Language	0.020 (−4.119 to 7.856)	0.540	0.008 (−14.694 to 16.438)	0.912	0.019 (−3.799 to 7.381)	0.530
Social self-help	0.011 (−3.769 to 5.205)	0.754	−0.135 (−21.288 to 0.446)	0.060	−0.015 (−5.184 to 3.094)	0.620
Total score	0.027 (−11.571 to 27.586)	0.422	−0.101 (−83.115 to 13.719)	0.159	0.004 (−16.792 to 3.094)	0.886
SRS scale scores	*n* = 912		*n* = 198		*n* = 1,110	
Social awareness	−0.078 (−6.353 to −0.566)	0.019	−0.013 (−6.782 to 5.615)	0.853	−0.065 (−5.478 to −0.243)	0.032
Social cognition	−0.057 (−7.227 to 0.485)	0.087	−0.133 (−15.714 to 0.379)	0.062	−0.066 (−7.356 to −0.411)	0.028
Social communication	−0.106 (−19.593 to −4.678)	0.001	−0.136 (−33.890 to 0.466)	0.056	−0.107 (−19.274 to −5.611)	<0.000
Social motivation	−0.058 (−8.188 to 0.496)	0.082	−0.030 (−12.326 to 8.004)	0.675	−0.049 (−7.298 to 0.670)	0.103
Autistic mannerisms	−0.045 (−8.622 to 1.557)	0.174	−0.074 (−17.647 to 5.418)	0.297	−0.050 (−8.582 to 0.688)	0.095
Total score	−0.090 (−45.386 to −7.307)	0.007	−0.109 (−75.921 to 9.442)	0.126	−0.090 (−43.795 to −9.113)	0.003
CARS scale scores	*n* = 924		*n* = 195		*n* = 1,119	
Total score	−0.106 (−15.360 to −3.738)	0.001	−0.211 (−36.867 to −7.157)	0.004	−0.126 (−17.000 to −6.182)	<0.000
Communication Warning	*n* = 785		*n* = 172		*n* = 957	
Behavior	−0.080 (−43.289 to −2.905)	0.025	−0.116 (−79.743 to 11.344)	0.140	−0.080 (−41.721 to −5.012)	0.013

*β (95% CI), regression coefficient (95% confidence interval).*

*Multivariate Linear Regression Models were employed to analyze the association between serum VA level and scores of scales, with age as covariant*.

### Associations Between VA and Neurodevelopmental Levels of Children With ASD in Different Sex Groups

The results in Table 5 showed that the effect of serum VA level was positively correlated with the general quotient (β = 0.082, 95% CI: 4.595 to 37.411, *P* < 0.05), gross motor quotient (β = 0.070, 95% CI: 2.058 to 39.480, *P* < 0.05), adaptive behavior (β = 0.066, 95% CI: 1.445 to 38.275, *P* < 0.05), language quotient (β = 0.089, 95% CI: 9.034 to 55.210, *P* < 0.05) and personal-social quotient (β = 0.085, 95% CI: 6.018 to 42.748, *P* < 0.05) in ASD children. We further analyzed the association between serum VA levels and development quotients in children of different sexes, and found that the serum VA level was positively associated with the general quotient (β = 0.073, 95% CI: 0.625 to 36.679, *P* < 0.05), gross motor quotient (β = 0.079, 95% CI: 2.599 to 43.533, *P* < 0.05), language quotient (β = 0.080, 95% CI: 3.391 to 54.324, *P* < 0.05) and personal-social quotient (β = 0.078, 95% CI: 2.009 to 42.391, *P* < 0.05) in boys with ASD. However, in girls with ASD, we only found that the VA level was positively correlated with the adaptive behavior quotient (β = 0.151, 95% CI: 3.835 to 97.569, *P* < 0.05).

**Table 5:** Effect of serum VA level on the scores of CBNS-R2016 in the ASD children at different gender groups.

**CNBS-R2016**	**Boys (*n* = 767)**	***P***	**Girls (*n* = 176)**	***P***	**Total (*n* = 943)**	***P***
	**β (95% CI)**		**β (95% CI)**		**β (95% CI)**	
Gross motor	0.079 (2.599 to 43.533)	0.027	0.032 (−38.166 to 58.457)	0.679	0.070 (2.058 to 39.480)	0.030
Fine motor	0.024 (−12.868 to 26.136)	0.504	0.069 (−23.753 to 62.839)	0.374	0.032 (−8.870 to 26.413)	0.329
Adaptive behavior	0.048 (−6.032 to 34.234)	0.170	0.151 (3.835 to 97.569)	0.034	0.066 (1.445 to 38.275)	0.035
Language	0.080 (3.391 to 54.324)	0.026	0.141 (−4.341 to 109.497)	0.070	0.089 (9.034 to 55.210)	0.006
Personal-social	0.078 (2.009 to 42.391)	0.031	0.104 (−14.632 to 18.023)	0.179	0.085 (6.018 to 42.748)	0.009
General quotient	0.073 (0.625 to 36.679)	0.043	0.120 (−7.931 to 74.712)	0.113	0.082 (4.595 to 37.411)	0.012

*β (95% CI), regression coefficient (95% confidence interval).*

*CNBS-2016R, Children Neuropsychological and Behavior Scale-Revision 2016.*

*Multivariate Linear Regression Models were employed to analyze the association between serum VA level and scores of CNBS-R2016 scale, with age as covariant*.

## Discussion

In this study, we conducted a nationwide multicenter cross-sectional survey focused on the serum VA levels in ASD children aged 2–7 years and their relationship with the core symptoms and neurodevelopmental level of ASD children of different sexes. Most studies, to date, have used small single-center samples to investigate the VA levels in ASD children, ignoring the influence of sex. Moreover, there is no relevant report on whether there are still sex differences in VA levels of children with ASD. To the best of our knowledge, this study is the first national cross-sectional survey to focus on the role of VA in sex differences in children with ASD in China. Our results highlight the need for sex stratification to investigate the effects of VA on core symptoms and neurodevelopment in children with ASD. We found that the effects of VA on core symptoms and neurodevelopment were more significant in boys with ASD, which may provide a new idea for the study of the mechanism of VA and ASD.

VA has been shown to be critical for cognitive development and normal neurological function of ASD patients. Several studies have reported VAD-related genetic and molecular abnormalities associated with children with ASD, including reduced serum levels of RA (26, 27), beta-carotene (28), and RALDH1 (29). We previously found that among children with ASD suffering from multivitamin deficiency, the VAD rate was the highest, at up to 77.9% (30) in Chongqing, China. These findings are consistent with the present study. Disordered or atypical eating patterns (including food refusal, diet restriction/pickiness, *etc*.) may have a causative role in serum VAD in ASD children (31). As reported (32) that serum VA levels in children with ASD were lower than those in TD children may be due to their specific dietary patterns, when ASD symptoms worsen, they may in turn aggravate abnormal eating behaviors in children with ASD, further exacerbating nutritional deficiencies (32, 33). However, such explanations do not fully explain the sex differences in VA levels in ASD children. We further performed stratified analysis by sex and found that, compared with the TD children group, the boys in the ASD children group had serum VAD, and VA levels in children with ASD were significantly lower in boys than in girls. Importantly, we previously observed a positive association between maternal micronutrient supplementation and VA levels in ASD children, and low serum VA levels in ASD children were associated with significantly lower rates of multivitamin supplementation during pregnancy, suggesting that the VAD may have started at the embryonic stage (19). For the prevention of ASD, it is necessary to balance nutrition during pregnancy and strengthen the prevention of VAD.

We also found that the serum VA level was negatively associated with the multiple subscale scores of SRS, including the SRS total score, the CARS total scores, and the scores of communication warning behavior of CBNS-R2016, which is consistent with the previous results of a small sample clinical study conducted by our research group (24). However, sex-stratified analysis was not performed in previous studies. In this study, as shown in Tables 3–5, we found that boys with ASD who had more severe clinical symptoms are more likely to have serum VAD. In addition, the serum VA level was positively correlated with the general quotient, gross motor quotient, language quotient, and personal-social quotient of boys with ASD, but no difference was found in girls. These findings indicate that there are sex differences in the effects of VA on clinical core symptoms and neurodevelopment in children with ASD. As is well known, ASD has a higher incidence (approximately 4.2:1) in males than in females (2), but the mechanism underlying the sex bias remains unknown. This led us to speculate that the effect of VA on the core symptoms and neurodevelopment of ASD children may be related to genetic and environmental factors.

VA can regulate central nervous system development through its active metabolite RA, which may regulate RORA through its RARs (34). As Valerie et al. reported, RORA may exhibit sex-related differences in gene expression in the human brain that are dependent on both brain region as well as stage of development (35). They also speculated that the involvement of RORA deficiency in the higher testosterone levels associated with increased risk for ASD. Another study reported that some children with ASD have mutations in RARα that disrupt downstream RA signaling, and fragile X mutations have also been found to impair RA signaling, as the fragile X mental retardation (FMR) protein interacts with RARα in RA-dependent homeostatic synaptic plasticity and homeostasis synaptic plasticity (36). Furthermore, RORA as a novel ASD candidate gene, which has been shown to be differentially regulated by male and female hormones, may contribute to sex bias in ASD (37). We previously found that RORA mRNA levels are significantly increased in children with ASD after the VA treatment (38). VAD caused by multiple factors during embryo or postnatal stage can lead to disturbance of RA signaling pathway. Therefore, the association between VA and ASD can be complex. As it is a cross-sectional survey, the causal relationship between VA and ASD cannot be determined. Further research is needed to confirm whether genetic or molecular abnormalities related to VA can lead to disruption of synaptic plasticity and affect the development of ASD and the sex bias. Thus, in addition to eating habits, we should also pay more attention to the sex bias of VAD, lifestyle and clinical manifestations in children with ASD and as well as to boys with ASD.

However, this study has several limitations that should be pointed out. Although this study is a national multicenter clinical survey, the causal relationship between VA and ASD cannot be determined as it is a cross-sectional survey. In addition, due to the large sample size of this study and the data collection from multiple cities, it is difficult to conduct a homogeneous dietary survey. Also, this study failed to collect data on dietary VA intake of ASD children. Moreover, although we found in this study that serum VA levels in ASD children were more significantly decreased in boys, and VA had a more significant effect on core symptoms and neurodevelopment in ASD boys, we only found such a clinical phenomenon and further mechanism research is necessary. More basic studies are needed to clarify the possible role of VA in sex bias in ASD children. Meanwhile, prospective cohort studies are necessary to explore the relationship between VA and the development of ASD, as well as prospective randomized controlled trials to further characterize the outcome of VA treatment.

In conclusion, we found that ASD children have lower serum VA levels than TD children, and the difference is more obvious in ASD boys. Serum VA status is associated with clinical symptoms and neurodevelopmental levels of ASD children, especially in boys with ASD. Therefore, we should pay more attention to the VA status of children with ASD and timely supplement as needed, particularly in ASD boys. Additionally, research should be conducted on the mechanisms of VA in ASD and its impact on ASD sex bias.

## Data Availability Statement

The original contributions presented in the study are included in the article/supplementary materials, further inquiries can be directed to the corresponding author/s.

## Ethics Statement

The studies involving human participants were reviewed and approved by Institutional Review Board of Children's Hospital of Chongqing Medical University. Written informed consent to participate in this study was provided by the participants' legal guardian/next of kin.

## Author Contributions

TY: conducted data collection and analysis, drafted, and revised the manuscript. TYL and JC: conceived and designed the research, revised the article, and conducted general supervision. LC, YD, FYJ, YH, LJW, XYK, MJY, JZ, LL, QH, JJC, SFF, YCW, QW, and CHJ: performed data collection, and analysis and interpretation. All authors contributed to the article and approved the submitted version.

## Funding

This work was supported by the National Natural Science Foundation of China (Nos. 81771223, 81770526, and 31971089) from TYL and JC, the Guangzhou Key Project in Early diagnosis and treatment of autism spectrum disorders (202007030002), the Guangdong Key Project in Development of new tools for diagnosis and treatment of Autism (2018B030335001) from TYL.

## Conflict of Interest

The authors declare that the research was conducted in the absence of any commercial or financial relationships that could be construed as a potential conflict of interest.

## Publisher's Note

All claims expressed in this article are solely those of the authors and do not necessarily represent those of their affiliated organizations, or those of the publisher, the editors and the reviewers. Any product that may be evaluated in this article, or claim that may be made by its manufacturer, is not guaranteed or endorsed by the publisher.
